# The Effect of Y on the Microstructure, Mechanical and Wear Properties of ZCuSn10Pb10 Alloy

**DOI:** 10.3390/ma15031047

**Published:** 2022-01-29

**Authors:** Zhaojie Wang, Guowei Zhang, Yuanyuan Kang, Yijun Liu, Xiaoyan Ren

**Affiliations:** 1 School of Materials Science and Engineering, North University of China, Taiyuan 030051, China; wzj1997vip@163.com (Z.W.); kangyy469627632@163.com (Y.K.); lyj15536626409@163.com (Y.L.); 2 Department of Mechanical Engineering, Taiyuan Institute of Technology, Taiyuan 030008, China; renxiaoyan03@126.com

**Keywords:** ZCuSn10Pb10, Y, lead particles, frictional wear, mechanical property, microstructure

## Abstract

We studied the effects of adding Y on the microstructure, mechanical properties and wear properties of ZCuSn10Pb10, and clarified the underlying mechanism by microstructure characterization through SEM, EDS and XRD. No new phase was detected after the addition of Y up to 0.2 wt.%, but an enrichment of Y in the Pb phase was found. The Pb particles were refined significantly after the addition of Y, which resulted from the compositional undercooling for the Cu dendrite where the Pb particles solidified, and the highest refinement efficiency was reached when the content of Y was 0.15 wt.%. The hardness of the alloy was improved due to the refinement of the microstructure. The fine Pb particles between the dendrite branches acted as solid lubricant, which was smeared on the entire surface during a friction and wear experiment, thus increasing wear resistance and reducing the coefficient of friction.

## 1. Introduction

Bronze is a copper-based alloy, with tin (Sn), lead (Pb) and zinc (Zn) as alloying elements [1]. Bronzes with Pb and Sn are widely used in industry. These alloys are used in shock loading applications such as piston pin bushings, rocker bushings, wear plates and thrust washers [2]. At the same time, lead-tin bronze has good wear resistance, high strength and elongation [3,4,5,6]. The wear resistance of the alloys is closely related to the inherent properties, such as strength, hardness, load, speed and lubrication [7,8]. Temperature will also affect the friction and wear properties through its effects on the thickness and composition of the deformed layer beneath the wear track or the contact surface properties [9].

Wear resistance and low coefficient of friction are some of the most important characteristics required for sliding bearings because one of the main mechanisms of wear is abrasive wear associated with sliding friction on the contact surface between the neck and the bushing. Without a lubrication film, direct contact between components can cause dry friction, which can lead to the wear and tear of bearings [10,11].

In lead-tin bronze, the microstructure consists of α-Cu solid solution, α-δ eutectoid [3,12] and lead phase in the copper substrate. When the Sn content is approximately 10%, lead-tin bronze usually has a typical dendritic structure, with large grains of α, and inter-dendritic α and δ eutectoid structure [13,14]. The soft Pb coating plays a good role in lubrication [15,16,17]. The distribution and morphology of lead particles show great effect on the friction performance of the alloy. Small and uniform lead particles are beneficial for the formation of a stable lubrication film on the friction interface to prevent dry friction between the two contact surfaces, thereby reducing wear rate [18,19].

The addition of rare-earth elements can purify Cu melts and improve microstructure, thereby improving the mechanical and other physical and chemical properties of Cu [20]. Adding trace rare-earth metals to cast lead-tin bronze can effectively prevent oxidation during the casting of lead-tin bronze, refine microstructure and improve wear resistance. However, studies of the friction and wear properties of the rare-earth-containing lead-tin bronze under service conditions are lacking [21]. Therefore, this paper explores the effects of the addition of Y on the microstructure, mechanical properties and friction and wear properties of lead-tin bronze. The evolution of mechanical properties and friction and wear properties are clarified.

## 2. Materials and Methods

### 2.1. Alloy Preparation

**Figure 1:**
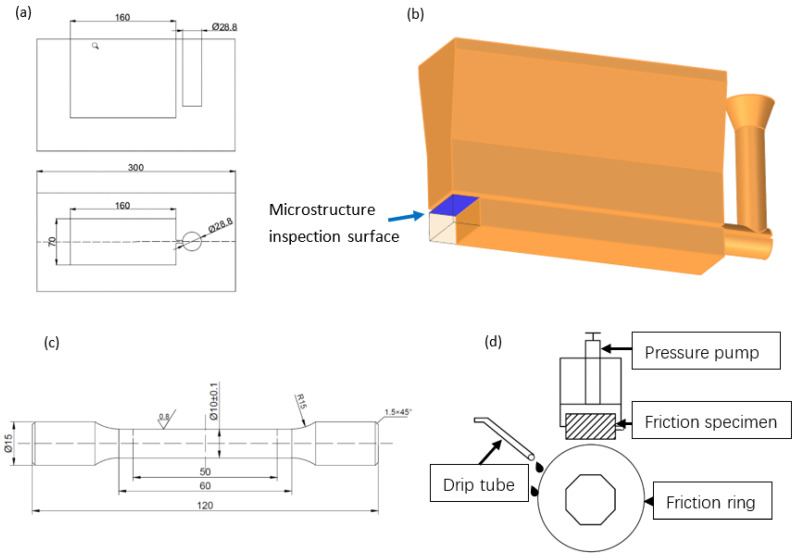
Schematic illustration of the (**a**) mold for casting, (**b**) cast specimen showing position for microstructure analysis, (**c**) tensile test specimen and (**d**) the contact mode of the friction pair.

Pure copper, pure zinc, pure lead, pure tin, pure nickel and copper-yttrium master alloy were used to prepare the Cu-10Sn-10Pb-1.75Zn-2Ni-xY (x = 0–0.2) (all compositions are in weight percentages). The pure copper and pure nickel were melted in a resistance furnace at 1150 °C, then the copper-yttrium alloy was added to the crucible, which was followed by pure zinc, pure lead and pure tin. The melt was then stirred with a graphite stick for composition homogenization. The melt with the temperature of 1150 °C was cast into a metal mold preheated to 250 °C, as shown in Figure 1a. The composition of the alloys was measured by atomic absorption spectroscopy.

### 2.2. Mechanical Property Test

Tensile test specimens with the dimensions shown in Figure 1a were prepared from the cast specimens, and tensile tests were carried out on a WDW-20/30 universal mechanical test machine. (SUNS, Shenzhen, China) The hardness of the specimen was measured using a HB-300B Brinell hardness machine (Huayin, Laizhou, China) with a load of 250 N and a dwell time of 12 s. Each sample was measured at least four times. A micro-hardness test was carried out with a load of 25 g and a dwell time of 10 s.

### 2.3. Microstructure Characterization

The specimen for metallographic characterization was cut from the cast piece, as shown in Figure 1b, ground by silicon carbide sandpaper up to 2000 grit, polished and etched with corrosion agent (hydrogen peroxide, ammonia, water, 1:1:3). The microstructure was characterized by a ZEISS optical microscope (ZEISS, Oberkochen, German) and a HITACHI SU 5000 field-emission scanning electron microscope (HITACHI, Tokyo, Japan) with the accelerating voltage of 20 kV under both secondary electron and back-scattered electron modes, and the element distribution was measured with a Bruker energy-dispersive spectrometer (EDS) (Bruker, Karlsruhe, German).

### 2.4. XRD Characterization

Samples for the phase composition analysis with the dimensions of 15 mm × 10 mm × 10 mm were cut from the same place as those for microstructure analysis, as shown in Figure 1b. They were ground by silicon carbide paper to 2000 grit and measured by X-ray diffraction with Cu Kα radiation under 40 kV and 40 mA in the Bragg–Brentano θ:2θ configuration on a Rigaku X-ray diffraction instrument (Rigaku, Yamanashi, Japan). The 2θ angle range was 10° to 90° with scanning speed of 4°/min.

### 2.5. Friction Wear Experiment

The friction test was carried out on an MRH-3A type high-speed ring block wear test machine (Yihua, Jinan, China); a schematic diagram of the friction and wear test mode is shown in Figure 1b. The loading force was applied to make the sample contact with the ring block, and lubricating oil was added to carry out high-speed friction movement. The material of the ring was 45# steel, and the frictional wear test was carried out at room temperature. The test method was low oil lubrication, and the lubricant used was 15W-40^#^. The speed of low oil lubrication was 1500 r/min with a test force load of 250 N and test time of 120 min. Before the test, all specimens were treated with the same procedure to ensure identical surface condition for all specimens: they were ground by silicon carbide paper, polished and cleaned in the ultrasonic cleaner for 5 min. The friction and wear results were the average of three tests. The sample and steel ring were cleaned with acetone using an ultrasonic cleaner after the test, then the specimens were weighed with an electronic analytical balance. The weight was measured five times and the wear rate was calculated as follows:

(1) $$\Phi =\frac{m_{1}-m_{2}}{\rho Fvt}$$

where: *Φ*, wear rate; *m*_1_, weight before wear experiment (g); *m*_2_, weight after wear experiment (g); *ρ*, density (g/cm^3^); *F*, test force (N); *v*, rotation speed (m/s); *t*, test time (s).

### 2.6. Density Measurement

The densities of the as-cast ZCuSn10Pb10 alloys were measured using the Archimedes method by immersing the samples in double-distilled water. The density of each alloy was the average of at least five measurements.

## 3. Results and Discussion

**Figure 2:**
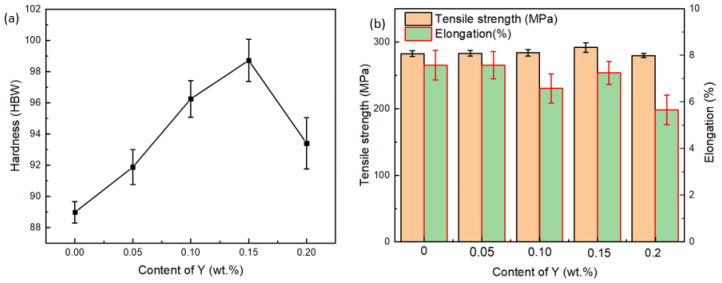
Evolution of (**a**) hardness and (**b**) tensile strength and elongation with increasing content of Y.

Figure 2a shows the evolution of the hardness of the ZCuSn10Pb10 alloy with the increase of Y content. There was a non-monotonic relationship between the content of Y and hardness of the alloys. With the increase of Y content, hardness first increased then peaked at a Y content of 0.15% with a hardness of 100.1 HBW, then decreased. The hardness of the 0.15Y-containing alloy was 13.5% higher than that of the ZCuSn10Pb10 alloy without Y.

There was no apparent change in the tensile strength of the ZCuSn10Pb10 alloy with increasing Y addition when the standard deviation of the experimental results were taken into account; as shown in Figure 2b, the tensile strength was around 293.2 MPa. The elongation of the alloy also varied little after adding Y.

**Figure 3:**
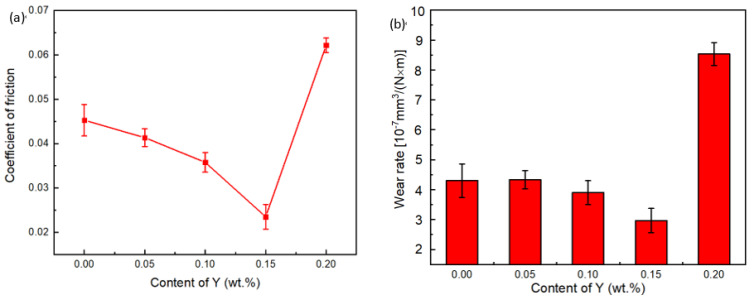
Evolution of the (**a**) coefficient of friction and (**b**) wear rate with increasing content of Y.

Figure 3 shows the evolution of the coefficient of friction and wear rate before and after the addition of Y, with the test force of 250 N, speed of 1500 r/min and the test time of 120 min. The coefficient of friction was approximately 0.045 without Y. After adding Y, the coefficient of friction decreased and then increased, and reached the minimum value when the Y content was 0.15%, with the minimum average coefficient of friction being 0.023. The wear rate is shown in Figure 3b. The evolution of wear rate exhibited a similar trend to that of the coefficient of friction with increasing Y content whereby the minimum value corresponded to the Y content of 0.15%, and the maximum value was reached after adding 0.2% Y.

**Figure 4:**
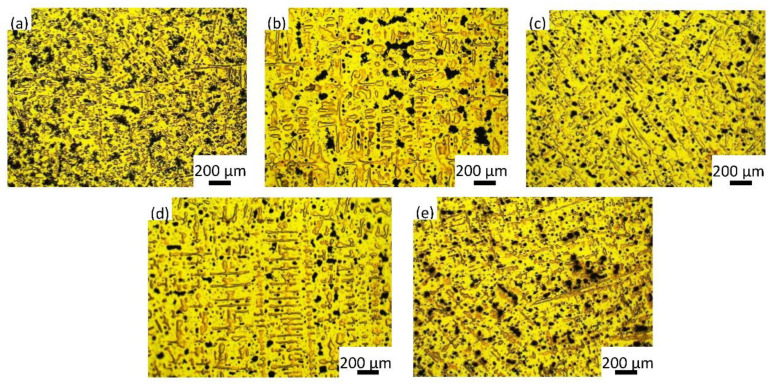
Optical microstructure of (**a**) ZCuSn10Pb10, (**b**) ZCuSn10Pb10 + 0.05% Y, (**c**) ZCuSn10Pb10 + 0.1% Y, (**d**) ZCuSn10Pb10 + 0.15% Y and (**e**) ZCuSn10Pb10 + 0.2% Y.

To investigate the origin of the evolution of the mechanical properties and friction and wear properties of the alloy with increasing Y content, the microstructure of the alloys was studied. Figure 4 shows that the as-cast alloys exhibited dendritic microstructures. The dendritic morphology was increasingly developed with increasing content of Y. The microstructure was also refined after the addition of Y due to the compositional undercooling developed in front of the solidifying Cu dendrite, and allowed the formation of more nucleate in the melt, which slowed the growth rate of the dendrite and led to finer, but developed, dendritic microstructure [20].

**Figure 5:**
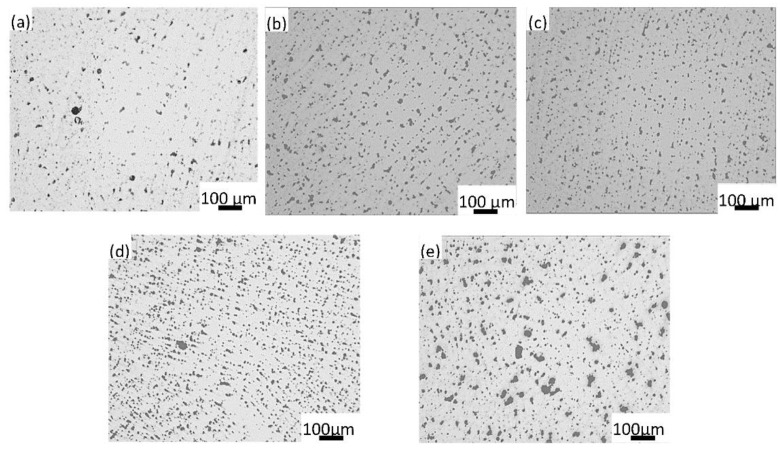
Optical microstructure showing the lead particles of (**a**) ZCuSn10Pb10, (**b**) ZCuSn10Pb10 + 0.05% Y, (**c**) ZCuSn10Pb10 + 0.1% Y, (**d**) ZCuSn10Pb10 + 0.15% Y and (**e**) ZCuSn10Pb10 + 0.2% Y.

**Figure 6:**
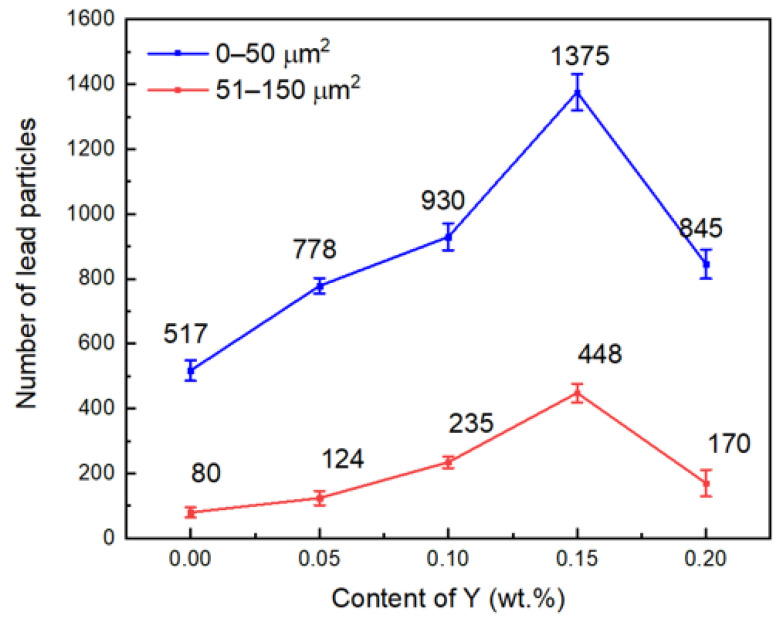
Changes in the number and size of lead particles with increasing Y content.

Figure 5 shows the lead particles. After adding Y, the lead particles of ZCuSn10Pb10 were refined, as can be seen from Figure 5a,d; it is obvious that when the content of Y was 0.15%, Y showed the highest efficiency of lead particle refinement, but when the Y content reached 0.2%, the refinement effect of lead particles was reduced. Figure 6 shows quantitatively that the number of lead particles in the range of 0–50 µm^2^ and 51–150 µm^2^ first increased and then decreased as the Y content increased. The refinement effect of the lead particles can be rationalized by the refinement of the dendrite. The finer structure divided the lead-containing liquid into smaller compartments, which refined the lead particles. However, when the Y content reached 0.2%, the lead particles coarsened. This may have resulted from the more developed Cu dendritic structure; the long dendritic branches may have contacted with each other earlier during cooling and formed larger compartments for the Pb-containing liquid phase that solidified into larger Pb particles. With excessive addition of Y, the temperature for the solidification of the Pb particles might also change and result in coarser Pb particles [20]. However, the exact mechanism underlying this phenomenon requires further study. The fine dendritic structure and lead particles play an important role in improving the mechanical properties of the alloy.

**Figure 7:**
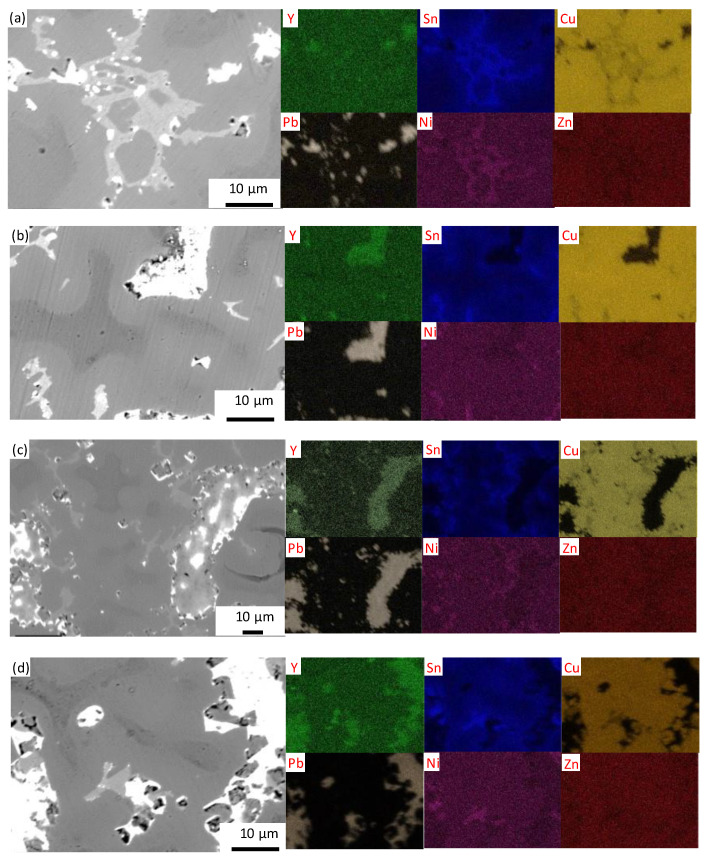
SEM micrograph (BSE mode) and EDS mapping of (**a**) ZCuSn10Pb10 + 0.05% Y, (**b**) ZCuSn10Pb10 + 0.1% Y, (**c**) ZCuSn10Pb10 + 0.15% Y and (**d**) ZCuSn10Pb10 + 0.2% Y.

The SEM results in Figure 7 show that there were three phases in the alloy. A lead-rich phase with the lightest contrast, a copper-rich phase with dark grey contrast, and discrete areas rich in copper and tin with light-grey contrast [3]. An enrichment of Y around Pb can be detected in Figure 7, which can also be seen in the EDS results of Table 1 where the content of the Y element in the Pb-containing phase was much higher than that around the α-Cu. This is because the solubility of Y in copper is very low, and the Y element segregates to the inter-dendritic region during solidification, where part of the Y element will be solidified with Pb.

**Figure 8:**
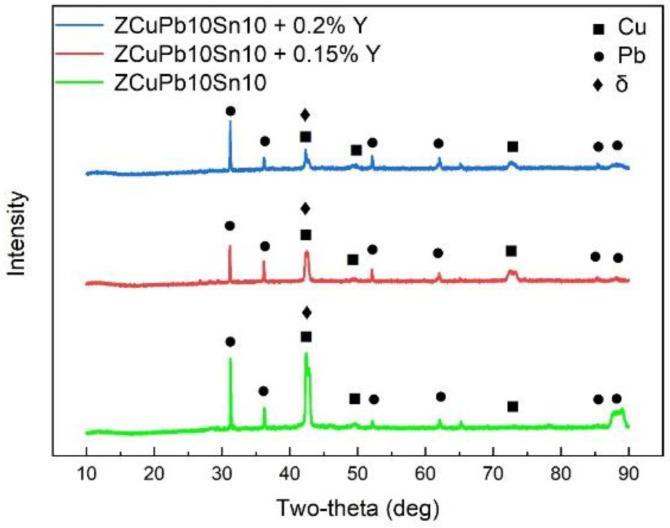
XRD results of the alloys with different Y contents.

To study the phase composition of the alloys with different Y content and identify the Y-containing Pb-rich phase, the XRD analysis was carried out and the results are shown in Figure 8. The diffraction peaks corresponding to the Cu, Pb and δ phases were detected for the alloys without Y [1] or with addition of Y, and no other phase was detected for the Y-containing alloys. Therefore, no new phase formed after the addition of Y. Y formed a solid solution in the Pb and Cu phase. To determine the effect of Y on the properties of Cu, Pb and δ phases, a micro-hardness test was carried out. The micro-hardness increased from 140 ± 7 HV to 176 ± 3 HV for Cu, decreased from 90 ± 4 HV to 53 ± 6 HV for Pb and remained relatively stable at around 137 HV for the δ phase after the addition of 0.15% Y. The increased hardness of the Cu matrix and the decreased hardness of the soft lubricant Pb contributed to the reduced coefficient of friction and decreased wear rate [22].

**Figure 9:**
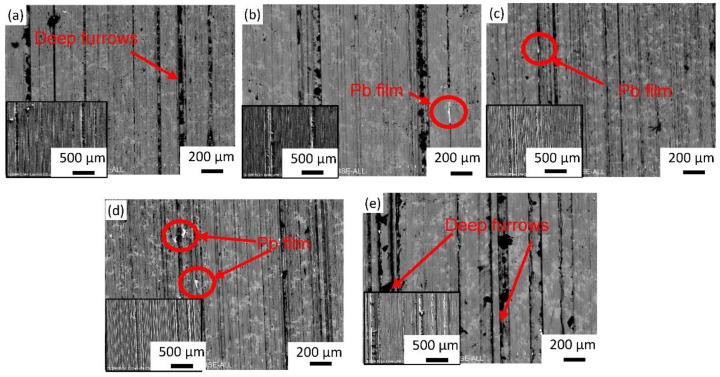
SEM micrograph (BSE mode) showing the surface after the wear test of (**a**) ZCuSn10Pb10, (**b**) ZCuSn10Pb10 + 0.05% Y, (**c**) ZCuSn10Pb10 + 0.1% Y, (**d**) ZCuSn10Pb10 + 0.15% Y and (**e**) ZCuSn10Pb10 + 0.2% Y, with inset (SE mode) indicating the relative depth of the wear track.

**Figure 10:**
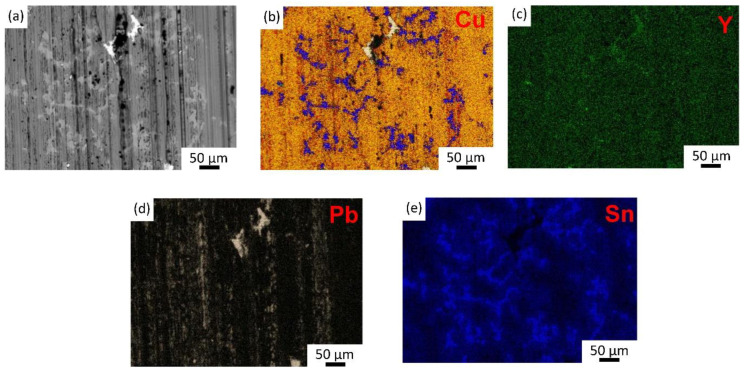
(**a**) BSE image and the EDS mapping results of (**b**) Cu, (**c**) Y, (**d**) Pb, and (**e**) Sn for the alloy containing 0.15% Y after the friction and wear test.

Figure 9 shows the surface of the wear-tested specimen with a load of 250 N and a speed of 1500 r/min. A very rough surface with a long and deep plow ditch and some sticky pits can be seen in Figure 9a. This likely resulted from friction surface contacts in the form of micro-convex structures, resulting in scratches and ploughs. Pb cannot be observed in the backscatter electron image, indicating that the original layer of Pb film on the surface was worn off. When Y was added, the depth of the plow ditch gradually became shallower, and the wear surface was more uniform; discontinuous Pb blocks and strips could still be observed on the surface after the friction and wear test, as shown in Figure 10, which enhanced the wear resistance of the material and reduced the coefficient of friction. Microstructure played an important role in the formation of the antifriction flat debris [17]. The decreased hardness of the Pb phase with the solution of Y element led to lower shear strength of Pb in the sliding direction, which was combined with a more homogeneous distribution of finer Pb particles. These are beneficial for the smearing of a homogeneous layer of Pb film on the mating interface. The increase in the micro-hardness of the Cu phase brought about higher resistance to plastic deformation of the matrix and prevented the formation of cracks, which is conducive to maintaining the Pb film on the interface and providing a good lubrication effect. [5]. However, when excessive Y was added, a large number of deep, long plow ditches and sticking pits appeared on the surface of the material as shown in Figure 9e, which indicates that with excessive Y addition, the large lead particles were very easily worn off during friction and wear tests. Pb soft lubrication film on the surface was also damaged and friction stability was lost, so the coefficient of friction and wear rate increased sharply.

The results above show that the effect of Y on the friction and wear properties depends on the microstructure evolution of the alloy with increasing Y addition. Both the hard matrix and the soft second phases evolved after adding Y. The refinement of the dendritic Cu structure and solution of Y strengthened the Cu matrix, leading to better load-bearing capacity. The addition of Y not only led to fine and homogeneously distributed Pb particles, but also reduced its strength, facilitating the formation of lead film during the friction and wear tests. All these changes in the microstructure help to preserve a good lubrication film on the interface. Since the refinement efficiency on both Cu dendrite and Pb particles peaked at a Y content of 0.15%, the ZCuSn10Pb10 alloy with 0.15% Y displayed the best friction and wear properties.

## 4. Conclusions

The effects of adding Y on the microstructure, mechanical properties and friction and wear properties of the ZCuSn10Pb10 alloy were studied, and the influencing mechanism of the Y on the alloy was clarified. The following conclusions are drawn:

Both the hardness and strength of the alloy first increased and then decreased with the increasing content Y, and the highest hardness and strength was reached when the content of Y was 0.15%.

Y refined both the dendritic structure and lead particles of the alloy, and the addition of 0.15% Y showed the highest refinement efficiency of lead particles.

No new phase formed after the addition of Y up to 0.2%. Y dissolved inside Pb, which increased the resistance of Pb to be worn off during the friction and wear test, and improved the friction and wear properties of the alloy.

## Figures and Tables

**Table 1:** EDS results of the point in Figure 7c (wt.%).

Point	Y	Pb	Sn	Ni	Cu	Zn
A	2.33	79.82	2.37	0.93	14.05	0.50
B	0.25	0.81	6.85	2.49	87.97	1.62

## Data Availability

The data are not publicly available due to it is part of another paper.
